# Emergence of verb-pattern morphology in young Arabic speakers: morphological and semantic features

**DOI:** 10.3389/fpsyg.2023.1127640

**Published:** 2023-05-12

**Authors:** Naila Tallas-Mahajna, Sharon Armon-Lotem, Elinor Saiegh-Haddad

**Affiliations:** ^1^Department of English Literature and Linguistics, Bar Ilan University, Ramat Gan, Israel; ^2^Al-Qasemi Academic College, Baqa al-Gharbiyye, Israel

**Keywords:** child language, Spoken Arabic, derivational morphology, verb patterns, roots, morphological complexity, semantic complexity

## Abstract

**Introduction:**

Arabic, a Semitic language, displays a particularly rich derivational morphological system with all verb stems consisting of a semantic root and a prosodic verb-pattern. Such regular and frequently encountered knowledge is expected to be acquired early. The present study presents a developmental perspective on the relative contribution of morphological and semantic complexity to the acquisition of verbs in Spoken Arabic.

**Method:**

Verbs in a spontaneous corpus from 133 typically developing children, 2; 6–6; 0-year-old, were coded for type and token frequency of verbal patterns and root type, and classified according to semantic complexity.

**Results:**

Results support an item-based emergence driven by semantic complexity at the earliest stages of acquisition. A developmental expansion in the diversity of verbal patterns and morphological complexity was observed with age. Morphological complexity is only identified when the same root appears in different verb patterns.

**Discussion:**

The late emergence of the same root in different verb patterns indicates that the perception of verb patterns as abstract linguistic entities beyond the actual verbs is attained later than the semantically-constrained verbs in earlier childhood. We conclude that whereas semantic complexity obstructs verbs from emerging in the lexicon in younger age groups, morphological complexity constitutes no such obstruction, since their perception as morphological devices is attained later in acquisition.

## 1. Introduction

Usage-based views of language development, both lexical and grammatical, argue that aspects of linguistic experience, and mainly frequency, “have an impact on the representation that is evidenced in speaker knowledge” (Bybee, 2006). Budwig's (1995) developmental-functionalist approach to child language proposes that children may neither be working verb-by-verb nor at an abstract rule level, but rather at some intermediate level by actively organizing what they take from the input into something that is more systematic and productive. The verb system in Semitic Languages in general and in Arabic in particular is often described as the conjugation of consonantal roots and templatic patterns. To examine the acquisition of such a morphological verb structure, we conducted a developmental study of verb acquisition in Spoken Arabic (a Palestinian Arabic dialect of the Muthallath area). The central focus of the study is on the actual role of morphological verb patterns vis-à-vis lexical-semantic complexity in the acquisition of verbs. Since linguistic units which are regular and frequently encountered are expected to be acquired early, our initial hypothesis was that children would resort to morphologically simpler verb patterns in earlier age groups and would gradually adopt verbs of more complex verb patterns in older age groups regardless of semantic complexity. This investigation is expected to shed light on the broader question of whether the traditional model involving verb patterns as derivational units holds for the emerging verb lexicon of young children, and at what chronological age. At the same time, the study will have implications for whether children in acquisition work “verb-by-verb” and when they start building up some more abstract root and pattern morphological structure representation.

Given the developmental-functionalist approach, it has been argued that children may neither be acquiring verb-by-verb nor at an abstract rule level, but rather at some intermediate level by organizing and systematizing the input into productive speech (Nelson, 1973; Budwig, 1995; Tomasello, 2003). Bybee (1995) argues that the likelihood of a morphological pattern being extended to new items in the lexicon depends on: (i) its defining morpho-semantic properties and (ii) its quantitative strength. According to Bybee, morphology is acquired based on raw lexical material of *type frequency* and its relative success and rate depend on the level of morpho-semantic complexity and on the quantitative input. As shown by Berman (1993), Hebrew-speaking children rely on the basic verb pattern, which also displays the highest type and token frequency, and from that pattern they develop the rest of the verb patterns (Berman and Dromi, 1984; Ashkenazi, 2015; Ashkenazi et al., 2016).

Verbs constitute the “architectural centerpiece” of grammar, as they determine the argument structure of the sentence (Golinkoff and Hirsh-Pasek, 2006). The gradient semantic complexity of verbs, which encode categories such as states, events, actions and processes, governs their order of acquisition (Gentner, 1978). Thus, the distinction between verb types such as stative *lie* and active *lay*, and also between intransitive *sit* and *rise*, on the one hand, and their transitive counterparts *seat* and *raise*, on the other hand, is not yet productive at the earliest stages of acquisition (Berman, 2004).

Gentner (1978) studied the order of acquisition of verbs and concluded (p. 996) that: “semantically simple verbs are learned before semantically complex verbs”, and since verbs label diverse semantic categories, they are more difficult to conceptualize by children than concrete nouns (Gentner, 1982; Golinkoff and Hirsh-Pasek, 2008). Children constantly construct meaning clusters comprised of semantic and pragmatic factors wherein they link forms with functions that meet their specific communicative needs. These meaning clusters serve as temporary solutions for children moving toward more adult-like constructions. Until they develop full command of these constructs, they use interim solutions including goal-blocking, i.e., mixing up transitive and intransitive verbs based on the ones that were acquired first, e.g., “the door won't open” (Cenko and Budwig, 2007). Moreover, children appear to approach verb learning in ways which vary between languages. This variation is based on specific structural characteristics of their native language. These specific characteristics pertain to differential typological organization of verb morphology and lexical semantics (Talmy, 1985; Kibrik, 2012; Koptjevskaja-Tamm, 2012).

Likewise, Berman (2004) insists on the methodological distinction between the *emergence* of a category and its *mastery* once acquisition has been achieved; based on this distinction, she asserts the relevance of the relative *semantic complexity* of each lexical item in the course of acquisition as a factor which determines the *order* of acquisition. The path from emergence to mastery evolves, and knowledge gradually consolidates “from semantically and structurally simplex and non-productive to more complex and productive” (Ravid, 2019). The modeling of the Hebrew verb morphology acquisition and increasing density of form-form relations as network of derivational families has recently been proposed by a novel methodology of Network Analysis (Dattner et al., 2022).

As a Semitic language, Arabic encodes the semantic relationship between verbs by morphological structure. The verbal system of Arabic consists of patterns which differ mainly in morpho-semantic class and in transitivity (Laks et al., 2019). Morphological affixes make patterns more complex, and the relative rise in morphological complexity allows the rise in semantic complexity, see Table 1.

**Table 1:** The verbal patterns in PA.

**Pattern**	**Example**	**Gloss**
CaCaC (or *fa'al*)	qasam	“Divide”
CaCCaC (or *fa”al*)	qassam	“Divide, split”
Ca:CaC (or *fa:'al*)	sa:ʕad	“Help”
ʔaCCaC (or *af'al*)	ʔaʕṭa	“Give”
tCaCCaC (or *tfa”al*)	tfarraj	“Look, observe (for a while)”
tCa:CaC (or *tfa:'al*)	tṣa:laḥ	“Make peace between”
inCaCaC^a^ (or *infa'al*)	infajar	“Burst”
iCtaCaC (or *ifta'al*)	i∫ta$\bar{g}$al	“Work”
iCaCC (or *ifa'all*)	iḥmarr	“Become red”
istaCCaC (or *istaf'al*)	istaʕmal	“Use”
CaCCaC (4) (or *fa'lal*)	daḥdal	“Roll”

^a^We do not include the spelling-biased customarily presented initial glottal stop in the actually /i-/ initial patterns as it is an epenthetic vowel with no preceding consonant, and the presence of the letter alif in the spelling is a graphemic convention free of any authentic phonemic reality in spoken Arabic This holds for the whole chapter.

This verb simple pattern *CiCeC*/*CaCaC* is considered the most basic verb pattern: *fa'al*; on the *scale of morphological complexity* this pattern is the simplest. It consists of two templates that vary in the vowel patterns: *a-a* and *i-e*. The *a-a* pattern *CaCaC* is more frequent in types and refers to an action performed by an agent; it can be either transitive (e.g., *katab* “write”) or intransitive (e.g., *qa*ʔ*ad* “sit”). The *i-e* pattern *CiCeC*, e.g., *xiser* “lose” functions as an inchoative verb (Laks et al., 2019). The *fa'al* basic pattern serves as a platform for additional morphological components, which make it more complex. The complexifying components vary between the doubling of a consonant, the lengthening of a vowel or the affixation of consonants or entire syllables. These additions to *fa'al* are called here *morphological affixes*, whereby each morphological affix makes the obtained pattern morphologically complex compared to the basic pattern.

These morphological affixes in the verb system, however, are far from insignificant language material. They often correspond to some enrichment of the meaning. The additional meaning components are called here *semantic affixes*. Correlation between morphological affixes and semantic affixes is best observed in non-singletons (Levie et al., 2020), that is in groups of verbs (two or more) which are derived from a single consonantal root, yet in different verb patterns. The relations between same-root verbs in different patterns are derivational and are manifested mainly in transitivity alternations and other types of semantic relations, like [±stative] or [±eventive] vs. [±agentive] and [±causative]; as well as in features like [±inchoative], [±reflexive], and [±reciprocal] (see Wittig, 1990; Fassi Fehri, 1994; Guerssel and Lowenstamm, 1996; Younes, 2000; Jastrow, 2004; Ryding, 2005; Hallman, 2006; Glanville, 2011; Ouhalla, 2014; among others).

A complementary question pertains to the role of morphological patterns is the prominence of the consonantal root. It has been shown that school children access lexical items in Hebrew and in Arabic by the mediation of the Semitic root (Ravid, 1995; Boudelaa, 2014; Shalhoub-Awwad and Leikin, 2016; Shalhoub-Awwad, 2020). Yet, these studies have studied adults or schoolchildren. No study, however, have yet addressed the status of the root in the verbal system of Spoken Arabic (henceforth SpA) during language acquisition before school age. We expect our study to demonstrate when children display positive signs of knowledge of the root as an independent linguistic unit.

The present study aims to examine the development of verb patterns in SpA in preschool children. More specifically, it sets as its goal to learn whether, and if so, identify at what stage of emergence, verbs cease to be used only lexically, and start being used as part of a complex morpho-semantic system. In other words, while the perception of verb patterns and roots is taken for granted in accounts of Arabic morphology, the current study aims to detect the chronological age at which verb derivational morphology becomes the speakers' cognitive reality. In order to address this question, we will measure the semantic and morphological parameters within the gradual emergence of the SpA verb system. The study will address the following research questions:

What is the developmental trajectory of emergence of verbal patterns in SpA?

a. Given that the influence of both morphological and semantic complexity develops with age, which of the two has a stronger impact on the emergence of verbal system?
b. What can the root-and-pattern structure of Arabic as a Semitic language inform us about the development of the verbal system from emergence to acquisition?

Based on previous findings from Hebrew, a typologically-similar language, it is predicted that in SpA too, morphologically simple verbal patterns will emerge in child speech before morphologically complex patterns and will gradually increase with age (Berman and Dromi, 1982, p. 420).

It is also predicted that semantically simple verbs will emerge before semantically complex verbs. Verb patterns differ in their distribution and frequency as types and tokens too, an extremely relevant factor for language acquisition as well (Gentner, 1978).

## 2. Materials and methods

### 2.1. Participants

A group of 133 preschoolers, divided into seven chronological age groups with 6-month intervals, from 2.6 to 6 years of age, participated in the study. All children attended nursery schools and preschool facilities in the vicinity of the Northern Triangle of Israel (around Kufur Qara', Ar'ara, Baqa-Jatt, and Umm al-Faḥm). All participant children are characterized by mid-high socioeconomic status (SES) and are native speakers of the central Israel Triangle dialects of SpA. All children are typically developing without any diagnosed psychological neurological or learning difficulties as reported by their parents. See Appendix A for more information regarding the demographics of the age groups targeted, including the number and gender distribution within each.

### 2.2. Tasks and procedure

Spontaneous child language samples were collected through elicitation tasks from each participant during sessions that lasted between half an hour and 1 h. All sessions were recorded. The data were collected individually by the first author in a quiet room within the kindergarten. Elicitation techniques included 10 pictures and posters which display situations related to everyday life of young children were chosen from a pamphlet of pictures, and the participant was asked to describe them, as well as a story retelling: children were read out-loud a well-known story of 430 words and had to retell it in their own words. It was made sure that the examined child would produce at least 100 utterances before the session was ended.

### 2.3. Data coding

Soon after the recording was completed, each language sample was transcribed using Arabic letters in dialect spelling. The examiner's utterances were also recorded and transcribed, and an accurate description of the recording context was added. All sessions, including the child's utterances, and the examiner's utterances were transcribed using phonetic transcription. An accurate description of the recording context was documented. In order to address the research questions, all verbs were extracted from the transcribed language samples yielding 253 verbs types. These verbs were then morphologically analyzed and sorted to 1 of the 11 verb patterns. Morphological complexity in the current study is determined by the number of morphological affixes in each pattern of the Arabic verb system; e.g., basic verb is *CaCaC qasam (CaCCaC), qassam (Ca:CaC qa:sam), (tCaCaC)*. *tqasam* Semantic complexity of verbs depicting features such as agentive vs. stative/eventive, and transitive vs. intransitive, as well as semantic properties of the verb: reflexive, causative, inchoative and reciprocate were coded.

All other verb patterns are morphologically-marked by at least one affix, e.g., root-consonant gemination, short-vowel lengthening, as well as different types of prefixation, infixation and circumfixation. These affixes mark each verb pattern as different along a morphological complexity scale, and their semantically-marked equivalents may convey various specific meanings, such as causative, reflexive, inchoative, etc. In what follows, we analyze the Arabic verb system based on this theory of affixes, both morphological and semantic; see Table 2 below.

**Table 2:** Morphological and semantic features of the verbal patterns of PA.

**Pattern**	**Morphological affix**	**Semantic affix/Function**
CaCaC	None	Various, unmarked
CaCCaC	Medial gemination	Causative
Ca:CaC^a^	Vowel lengthening	Conative^b^
ʔaCCaC	ʔa- prefix	Causative
tCaCCaC	t- prefix + medial gemination	Reflexive/medio-passive
tCa:CaC	t- prefix + vowel lengthening	Reciprocal
inCaCaC	in- prefix (n- prefix)	Medio-passive
iCtaCaC	i+ta circumfix (-ta- infix)	Reflexive intransitive
iCCaCC	Final gemination	Inchoative of colors
istaCCaC	ista- prefix (sta- prefix)	Reflexive transitive
CaCCaC (4)	Additional medial consonantal slot	No semantic affix

^a^The vowels between the root consonants, which appear in the basic pattern CaCaC, may become prosodically unnecessary and drop, as in this pattern: ʔaCCaC (not ʔaCaCaC ʔa- prefix). For a theory on the prosodic constraints on the Semitic verb and nouns patterns, see McCarthy (1981).

^b^The conative function: engages the Addressee (receiver) directly and is best illustrated by vocatives and imperatives, e.g., “Tom! Come inside and eat!”

### 2.4. Data analysis

All analyses were conducted in R version 4.0.5. (Team and R Core Team, 2012). To analyze the effect of age on the tokens across verbal patterns, a Linear Mixed Models (LMM) analysis was run with Participants and the random effect and age group, pattern and the age group X pattern interaction and the fixed effects. In this and all other mixed models analyses, we began with the model which included only the dependent variable and the random factor, then adding fixed factors. The maximum likelihood estimations were applied to test the significance of each model using Chi square distributions. All interactions were examined using plots from the *sjPlot* package (Lüdecke, 2021). *Post-hoc* analyses were applied using the Estimated Marginal Means package *emmeans* (Lenth, 2019).

To analyze the effect of age across verbal patterns on types, a Generalized Linear Model (GLM) analysis with Poisson distribution was performed. Since verbal types were coded per pattern (rather than per child), we used raw frequencies as the dependent variable for the analysis of pattern types. This allowed us to demonstrate the increasing use of the variety of patterns. The Poisson distribution was applied which is the best fit for the frequency (count) data.

For the analyses of agentivity, transitivity, and of semantic classes, Generalize Linear Mixed Models (GLMM) analyses with binomial distribution were used, where for each verb item its use in each age group and its semantic status were coded. The random factor in these analyses was verb items and the fixed factors were age group, verbal type (agentive/stative, transitive/intransitive, and causative/reciprocal/reflexive/inchoative, respectively), and their interaction. The binomial distribution was applied, since for each verbal items its use (1) or lack of use (0) was coded.

## 3. Results

### 3.1. The developmental trajectory of the emergence of verbal patterns

To describe the developmental trajectory of the emergence of verbal patterns in SpA and to answer the question of whether morphologically simple verb patterns emerge before morphologically complex patterns, we counted the number of tokens and types within each verb pattern. Descriptive statistics are presented in proportional use of each pattern per age group.

#### 3.1.1. Verbal patterns—tokens

**Figure 1:**
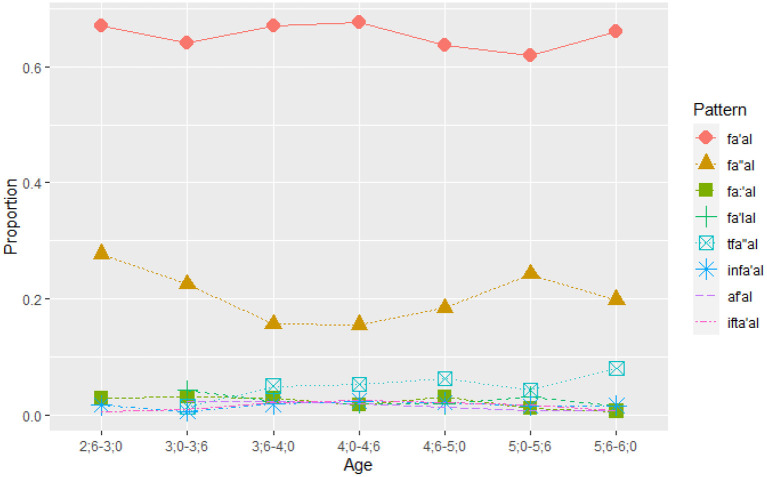
Proportion of verb *tokens* per age group^a^. ^a^The verb patterns *tfa:'al* and *istaf'al* figure in the corpus in negligible numbers, and are not presented in the results.

Figure 1 presents the proportion of verb tokens in each pattern within each age group, showing the developmental changes in the distribution of verbs across patterns.

Figure 1 illustrates the proportion of tokens used in each verb pattern in each age group. It shows that two patterns *fa'al* and *fa”al* were most frequently used by the youngest age group (2.6–3), while none of the children in this group used in *af'al, tafa:'al, istaf'al, flala*. Two more emerge by the next group (3–3.6), *fa:'al* and *fa'lal*, while from 3.6 onwards all other patterns are used. For all age groups, *fa'al* and *fa”al* are the most frequently used patterns with verb tokens in *fa'al* being more than 60% of the verb tokens used, regardless of the increase in the number of verb tokens as age goes up. It further shows that the variation in other verb tokens does not change the dominance of *fa'al*, and the increase is rather at the expense of *fa”al*.

LMM analysis was used to test the effects of Age, Pattern, and the Age x Pattern interaction on the proportion of verb tokens in each pattern. The analysis revealed a significant effect of Pattern, χ^2^ = 3879.15, *p* < 0.001 (AIC = −216.0), and a significant interaction of Age X Pattern, χ^2^ = 181.87, *p* < 0.001 (AIC = 3,403.4). The effect of age was not significant χ^2^ = 9.52, *p* = 0.15 (AIC = −3507.2). Model's estimates, CI, and *p*-values are included in Appendix B.

*Post-hoc* analysis of the interaction using *emmeans* function with the Tukey method of correction revealed that for *fa'al* there was a significant difference between 4.0 and 4.6 (*p* = 0.05) and 5.0 and 5.6 (*p* = 0.04); for *fa”al*, there was a significant decrease from 2.6 to 3.0 (*p* = 0.004), from 3.0 to 3.6 (*p* < 0.001), and increase from 4.6 to 5.0 (*p* < 0.001), and decrease from 5.0 to 5.6 (*p* = 0.02). For *fa:'al, fa'lal, tfa”al, infa'al, af'al*, and *ifta'al* there were no significant differences between age groups. Thus, overall, an increase was observed only for *fa'al* at 5 years and for *fa”al* at 3 and 5.

#### 3.1.2. Verbal patterns—types

**Figure 2:**
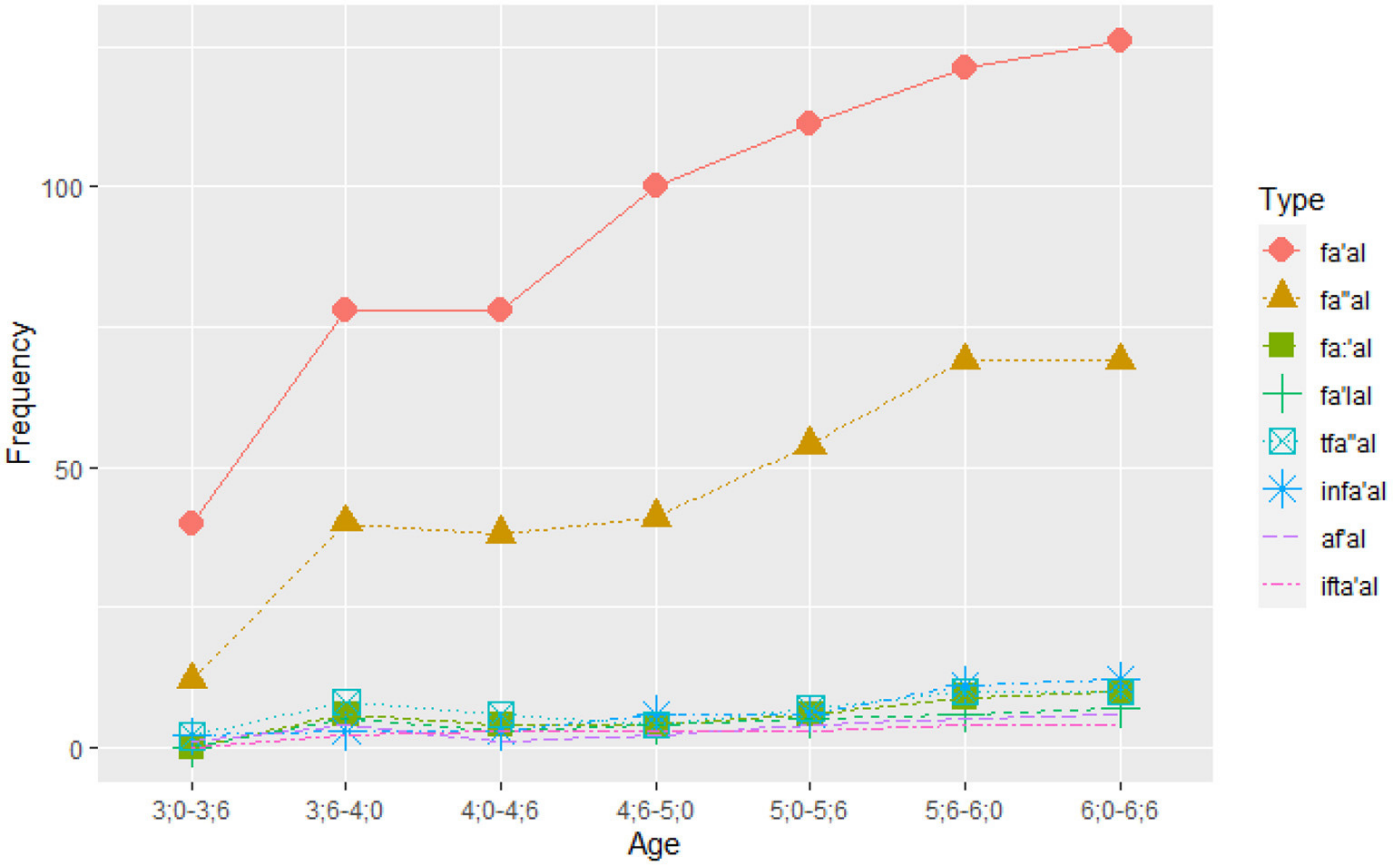
Frequencies of verb *types* per age group.

Figure 2 illustrates the proportion of types used in each verb pattern in each age group. It indicates that two patterns *fa'al* and *fa”al* were most frequently used by the youngest age group (2.6–3). For all age groups, *fa'al* and *fa”al* are the most frequently used patterns with verb types in *fa'al* being more than 60% of the verb types used, regardless of the increase in the number of verb types as age goes up. It further shows that the variation in other verb types does not change the dominance of fa'al, and the increase is rather at the expense of fa”al. Two more verb patterns emerge by the next group (3–3.6), fa:'al and fa'lal, while from 3.6 onwards all other patterns are used.

GLM was used to tests the effects of Age, Pattern, and the Age x Pattern interaction on the frequency of verbal pattern types. The analysis was performed on frequencies in order to trace the increase in use of types per age group and pattern. A significant main effect of Age emerged, χ^2^ = 1.68, *p* < 0.001 (AIC = 2,129.90), and a significant main effect of Pattern, χ^2^ = 2,043.88, *p* < 0.001 (AIC = 268.46). The interaction of Age and Pattern was not significant (*p* < 0.99) (AIC = 326.67). The optimal model then included the Age group and the Pattern factors. Appendix C shows the model summary with Incidence Rate Ratios, CI, and *p*-values. The model had *R*^2^ (Nagelkerke) of 1.00.

Since the GLM analysis uses dummy coding where the comparison group was set to 2.6–3.0, Appendix C lists the comparison to one reference category. We applied *post-hoc* analyses using the *emmeans* package with Tukey corrections in order to compare developmentally adjacent age groups. The *post-hoc* analyses reveled that there was a significant increase only between the age groups of 2.6–3.0 and 3.0–3.6 (*p* < 0.001). For effect of Pattern, *fa'al* was the reference category in the analysis. We ran *post-hoc* analyses using Tukey corrections which revealed that *fa'al* was the most frequently used pattern (*p* < 0.001). In addition, *fa”al* was used more frequently than all other categories (*p* < 0.001). The difference among other patterns was not significant.

### 3.2. Emergence of verb semantics features

Whether semantic complexity or morphological complexity determines the order of emergence, the verb types were classified first according to the feature [±agentive], next by transitivity [±transitivity], and finally by semantic verb class focusing on [+causative], [+reciprocal], [+reflexive], and [+inchoative]. The category of verb pattern was not included in the analysis since the same pattern with a different root can be classified in different manners, and the pattern remains the same, e.g., both infa'al as in 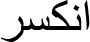/inkasar/“break” (intransitive) and infa'al as in 
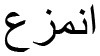
/inmazaʕ/“be torn” (passive).

#### 3.2.1. Agentivity

A verb is analyzed as [+agentive] if the individual is involved in an action which entails change from state A to state B. Agentive verbs have more complex meanings, i.e., a higher number of entailed properties. The rationale for this hypothesis is that understanding a stative verb requires the activation of a single non-dynamic situation (Gennari and Poeppel, 2003). Conversely, a verb is analyzed as [–agentive] if the individual is not involved in an action of change and the verb is thus [+eventive], or if there is no change and the verb is thus [+stative]. According to the feature of semantic properties [*agentive*] vs. [*stative/eventive*], Table 3 shows the percentages of *agentive* vs. *stative/eventive* verb types per age group. For all age groups, the percentage of agentive verb types is the largest.

**Table 3:** Agentive vs. stative/eventive verb types by age group (in percentages).

**Age**	**2.6–3**	**3–3.6**	**3.6–4**	**4–4.6**	**4.6–5**	**5–5.6**	**5.6–6**
Number of verb types/semantic properties	57	146	136	165	200	239	248
Agentive	84%	92%	89%	88%	88%	88%	88%
Stative\eventive	16%	8%	11%	12%	12%	12%	12%

A GLMM analysis with binomial distribution was performed on the production of verbal patterns across seven age groups. The following fixed factors were tested: Age group, Agentivity [±agentive], and Age ^*^ Agentivity. The analysis revealed a significant effect of Age group, χ^2^ = 252.24, *p* < 0.001 (AIC = 4,257.8), a significant effect of Agentivity, χ^2^ = 1,291.80, *p* < 0.001 (AIC = 2,968.0), and a significant interaction of Age group ^*^ Agentivity, χ^2^ = 27.13, *p* < 0.001 (AIC = 2,952.9). Appendix D shows the summary of the optimal model including the interaction of Age group and of Agentivity.

In order to understand the interaction, the predicted odds of producing verbal pattern types of [±agentive] were plotted based on the model using the *sjPlot* function and *post-hoc* analyses were conducted using the *emmeans* function.

**Figure 3:**
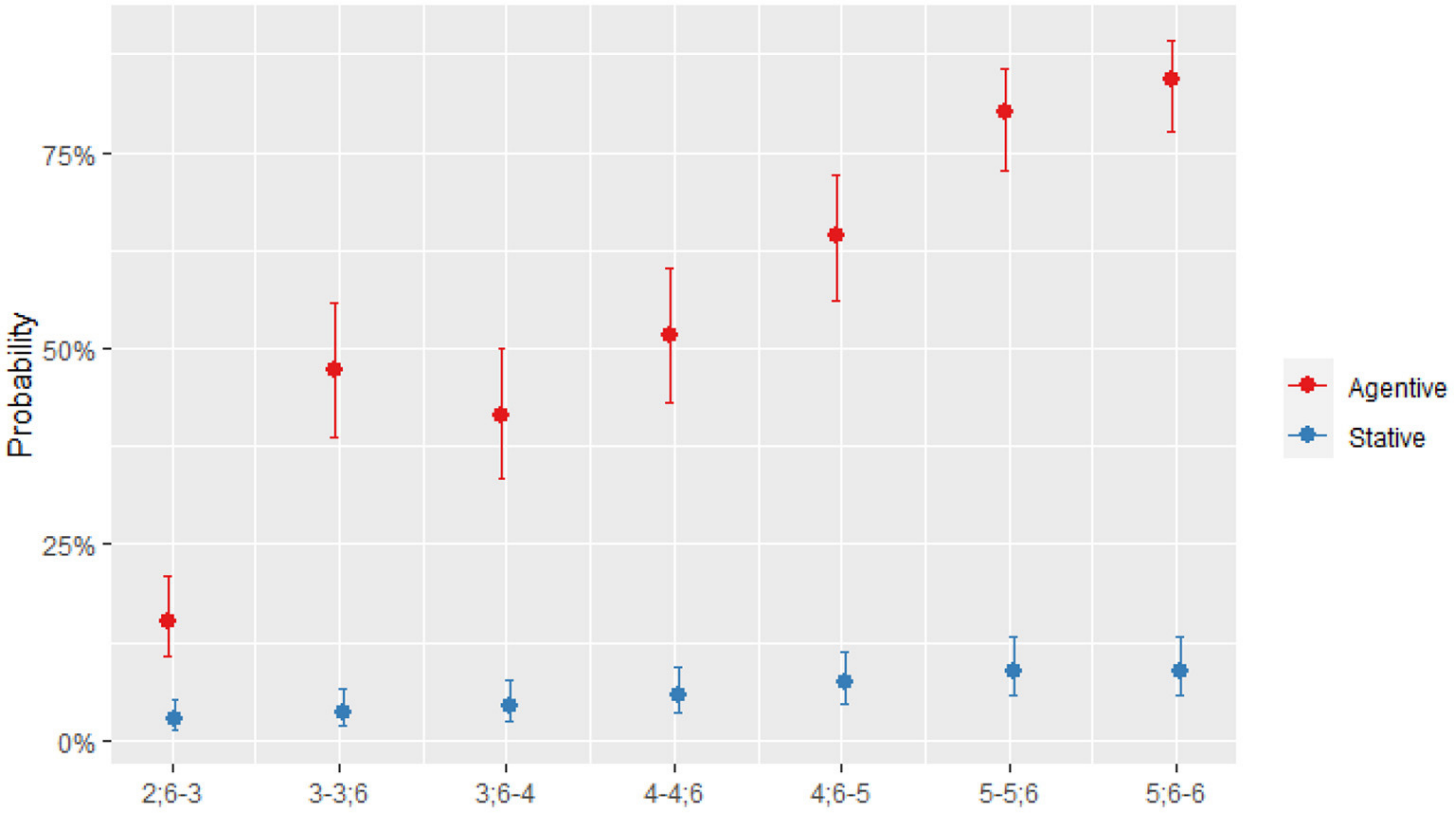
Predicted probability of production agentive/stative verb types.

Figure 3 shows that while the use of stative verbs remains the same across age groups, increase in the number of agentive verbs used occurs mainly at the age of 3. *Post-hoc* analyses applying Tukey corrections revealed that a significant increase occurred at age 3 (*p* < 0.001), further increase also occurred at 4.6, but did not reach significance (*p* = 0.07), and at the age of 5 (*p* = 0.006). The differences between other developmentally adjacent age groups were not significant. The increase was observed only for agentive verbs.

#### 3.2.2. The emergence of verb semantics—transitivity

We further classified the verbs according to the feature of semantic of transitivity [±transitive] and show the percentages of transitive and intransitive verb types per age group (see Table 4).

**Table 4:** Transitive and intransitive verb types by age group (in percentages).

**Age**	**2.6–3**	**3–3.6**	**3.6–4**	**4–4.6**	**4.6–5**	**5–5.6**	**5.6–6**
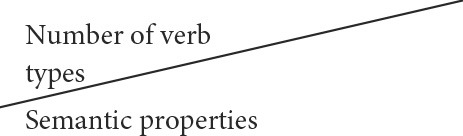	57	146	136	165	200	239	248
Transitive	39%	55%	59%	58%	58%	58%	58%
Intransitive	61%	45%	41%	42%	42%	42%	42%

While for the youngest age group there were more intransitive than transitive verbs, this pattern is reversed by the age of 3.

A GLMM analysis was performed on the use of transitive/intransitive verb types (coded as a dichotomous variable) with Age, Transitivity [±transitive], and Age × Transitivity interaction as explanatory variables. The analysis revealed a significant effects of age group, χ^2^ = 241.28, *p* < 0.01 (AIC = 4,189.6), a significant effect of Transitivity, χ^2^ = 48.68, *p* < 0.01 (AIC = 4,142.9), and a significant interaction of Age and Transitivity, χ^2^ = 18.18, *p* = 0.006 (AIC = 4,136.7). Appendix E shows the optimal model's estimates.

**Figure 4:**
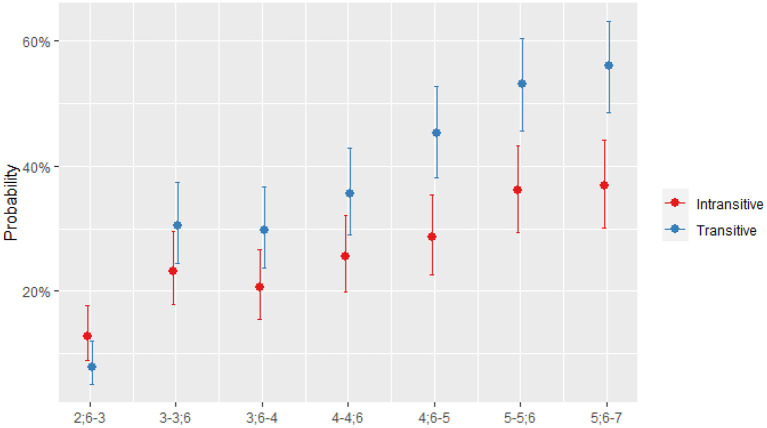
Predicted probability of using verb types across age groups.

To capture the meaning of the main effects, Figure 4 displays the predicted frequencies of using transitive and intransitive across all age groups.

Figure 4 shows that in the youngest age group intransitive verb types were most frequent, but starting from age 3 transitive verb types are predicted to be used with higher frequency. *Post-hoc* analyses with Tukey corrections showed that in age groups 2.6–3 (intransitive > transitive) and 3–3.6 (transitive > intransitive) the difference between ± transitive almost reached significant (*p* = 0.06 in both age groups), starting from age 3.6 transitive types are predicted to be used significantly more frequently in each older group (*p* = 0.02, *p* = 0.01, *p* < 0.001, *p* < 0.001, *p* < 0.001, respectively).

#### 3.2.3. The emergence of verb semantics—semantic classes

Verbs types were classified into semantic classes according to the feature of [+causative], [+reciprocal], [+reflexive], and [+inchoative]. Table 5 presents the percentage of verb types in each semantic class by age group, showing that inchoative verbs were most frequent followed by causative verbs, reflexive verbs and reciprocal verbs, in this order.

**Table 5:** Semantic class [+causative], [+reciprocal], [+reflexive], and [+inchoative] by age group (in percentages).

**Age**	**2.6–3**	**3–3.6**	**3.6–4**	**4–4.6**	**4.6–5**	**5–5.6**	**5.6–6**
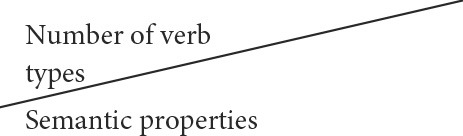	57	146	136	165	200	239	248
Inchoative	77%	62%	57%	61%	59%	59%	59%
Causativity	14%	31%	33%	31%	33%	36%	34%
Reflexivity	9%	8%	9%	7.00%	7%	7%	7%
Reciprocity	0%	0.60%	0.70%	1.00%	2.00%	1%	1%

A GLMM analysis was performed on the probability of verb types as a function of Age group, Class ([+causative], [+reciprocal], [+reflexive], and [+inchoative]), and Age ^*^ Class interaction as explanatory variables. The analysis revealed a significant effect of Age group, χ^2^ = 205.27, *p* < 0.001 (AIC = 6,194.0), Class, χ^2^ = 1,413.6, *p* < 0.001 (AIC = 4,786.4). The interaction of Age group and Class was not significant, χ^2^ = 17.32, *p* = 0.50 (AIC = 4,805.1). Appendix F shows the optimal model's estimates including the main effects of Age group and Class. Pairwise comparisons for Class using Tukey corrections indicate that children used more [+inchoative] than [+causative] (*p* < 0.001), [+reciprocal] (*p* < 0.001), and [+reflexive] (*p* < 0.001) verbs, and the hierarchy between the four classes was significant across all age groups.

### 3.3. Root-pattern emergence

The third sub-question asks whether the emergence of verb patterns is related to the emergence of specific roots, or alternatively, roots and patterns emerge independently of each other in Spoken Arabic. To address this question, a qualitative approach was used as we identified roots that appeared in more than one pattern in each age group. The corpus under investigation contained alongside roots that appear in one pattern (singletons) also some roots that appeared in more than one pattern (non-singletons). A total of 252 verb types were produced from the conjugation of 220 roots with the verbal patterns attested in the corpus. That is, only 30 roots were conjugated in more than one verbal pattern. Table 6 presents the use of these non-singletons at least once by age group.

**Table 6:** Emergence of non-singletons per root by age-group.

**Verb non-singleton (pair)**	**Age group**						

	**2.6–3**	**3–3.6**	**3.6–4**	**4–4.6**	**4.6–5**	**5–5.6**	**5.6–6**
sabaḥ/tsabbaḥ (swim/swim)	+	+	+	+	+	+	+
ḥammam/tḥammam (make someone take a shower/take a shower)	+	+		+		+	+
ḡasal/ḡassal (wash/wash continuously)	+			+		+	+
xa:f/xawwaf (fear/frighten)		+	+	+	+	+	+
xabba/txabba (hide something or someone/hide himself)		+	+			+	+
xarraf/txarraf (talk/tell)		+	+			+	+
ḍiḥik/ḍaḥḥak (laugh/make someone laugh)		+	+		+	+	+
wiqiʕ/waqqaʕ (fall/make someone fall)		+	+		+	+	+
∫irib/∫arrab (drink/to make someone drink)		+	+	+		+	+
kasar/ankasar (broke/was broken)		+		+	+	+	+
wiṣil/waṣṣal(arrive/deliver)		+		+	+	+	+
masaḥ/massaḥ (wipe/wipe continuously)		+			+	+	+
rijiʕ/rajjaʕ (come back/ bring back)		+			+	+	+
waqqaf/awqaf (stop/stop something or someone)		+				+	+
∫irif/t∫arraf (know/get to know)		+				+	+
∫atta/a∫ta (rain/rain)		+				+	+
fa:t/fawwat (enter/put something or someone in)			+	+	+	+	+
naqal/naqqal (transfer/transfer continuously)			+	+	+	+	+
ra∫aq/ra∫∫aq (splash/splash continuously)			+		+	+	+
libis/labbas (wear/dress someone)				+	+	+	+
liʕib/la:ʕab (play/make someone play)				+	+		+
∫a$\bar{gg}$al/ishtaḡal (make someone or something work/'work)				+		+	+
qaṭaʕ/anqaṭaʕ (cut/be cut)				+		+	+
xalla/txalla (make something or someone stay or let (do)/give up on)					+	+	+
kabb/inkabb (pour/be poured)					+	+	+
dabb/indabb (push to the ground/be pushed to the ground)					+	+	+
qatal/tqa:tal (fight someone/fight with someone)					+		+
ʕimil/istaʕmal (make/use)						+	+
ḡiriq/ḡarraq (drown/make someone drown)						+	+
xirib/xarrab (be damaged /cause damage)						+	+
Total	3	15	10	13	17	28	30

Three non-singletons appeared already in the youngest group 2.6–3 (1.19% of a total number of all verbs, 5.2% of the verb types used by this age group) and a transition was observed in the next age group with 15 such cases (6% of all verbs, 10.3% of the verb types used by this age group), and up to 30 non-singletons (12% of all verbs, and of the verb types used by this age group) in the oldest age group (5.6–6). That is, the growth in the number of non-singletons beyond the age of 3 is compatible with the increase in the number of verb types, with some non-significant fluctuation between age groups.

## 4. Discussion

A corpus of 1,907 Spoken Arabic verb tokens in 252 verb types collected from a sample of 133 children aged from 2.6 to 6.0 generated quantitative data of the distribution of *the different verb patterns* and their differential usage across seven age-groups. These data were used to explore the developmental trajectory of the emergence of verbal patterns in SpA, focusing on the role of pattern derivational morphology of verbs, semantic properties and roots in this process.

### 4.1. Morpho-semantic complexity in the emergence of Arabic verbs in the early years of life

The developmental trajectory of the distribution of verb patterns by age group in SpA showed that the morphologically simplest verb pattern *fa'al*, is both the first pattern to emerge and the most frequent pattern in use throughout all age groups. The second verb pattern to appear in the first age group is the *fa”al* (geminate) pattern which is the next in complexity and is less simple than the morphologically simplest. In fact, this pattern was also represented in the youngest age group by verbs such as ḥ*ammam* “wash someone's body” and *kayyaf* “have fun”. Since the medial root-consonant geminate verb pattern is not the simplest one, and still, it appears at the youngest age group, one might ask how it is possible for young children to display such morphological complexity. The answer resides in the significant role of consonant gemination in the rest of the lexicon of Spoken Arabic. The fact that many Arabic nouns, e.g., '*immi* “my mother” display consonant gemination makes this property an inherent phonemic feature to the entire lexicon of Arabic, not only to the verb system, and therefore, this relatively complexifying component of the verb pattern *fa”al* is not phonologically too complex for Arabic-speaking children in the developmental trajectory of the verb system. In other words, since consonant gemination is phonemic in Arabic also outside the verb system, it is semantically contrastive within the verb system contributing to its early emergence. With respect to the developmental trajectory of the verb system, the results show that the older age groups display more complex and more diverse verb patterns. At age 3–3.6 more complex verb patterns emerge than in the previous age group, but very scarcely. From age 3.6 on we observe the remaining verb patterns in a more noteworthy proportion, and this leads to a greater morphological diversity in the verb system. The more complex verb patterns gain in proportion of verb types as the children get older.

Turning to *semantic complexity*, our results confirm that the early emergence of verbs is determined by their semantic properties, primarily by agentivity and transitivity. Our results reveal that although both agentivity and transitivity make verbs semantically more complex, only transitivity grows significantly from age 3 to 3.6 on, and increases with age, whereas agentivity does not. We therefore argue that the semantic complexity gap between [–agentive] events and [+agentive] actions is less significant than the complexity gap between [–transitive] states or actions involving only one argument, usually referred to as the subject, and [+transitive] actions which necessarily involve additional arguments, usually referred to as object(s) or verb complement(s). For example, as early as in the age group 2.6–3 semantically simple no-object verbs emerged such as *na:m* “sleep”, *inbasat* “enjoy”, *ra:ḥ* “be away” and *li*ʕ*ib* “play”. Also, the effect of age is such that children used more inchoative verbs than causative verbs specifically after the age of 3, because while causative verbs involve a greater number of arguments, inchoative verbs convey the beginning of a state or an activity, a sense which requires nonetheless some perception of time.

**Figure 5:**
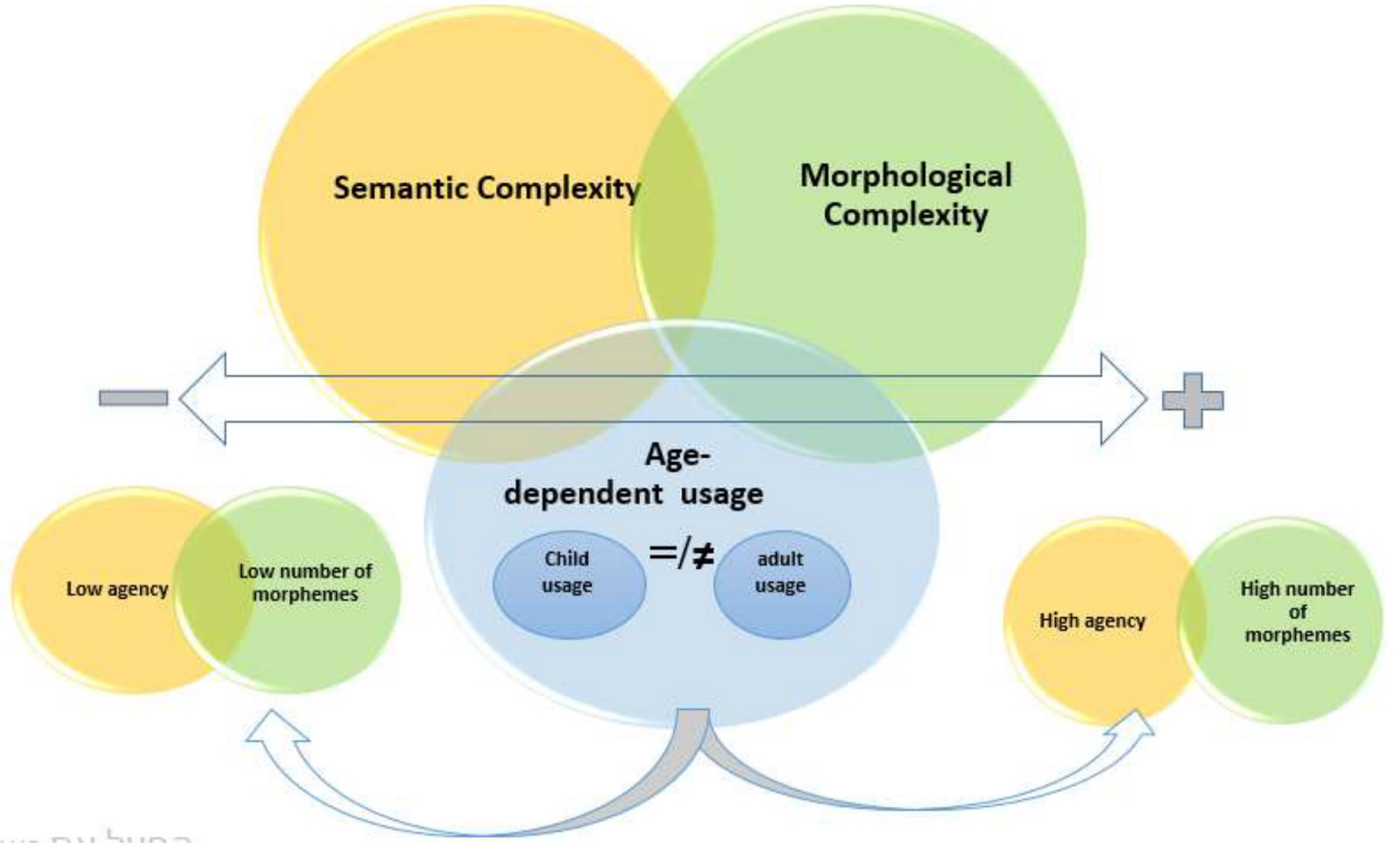
Age-dependent usage-based *rising* semantic and morphological complexity.

In the next age group 3–3.6 slightly more semantically complex verbs emerge, and this leap involves causative verbs entailing one additional argument, e.g., *xawwaf* “scare”, *xabba* “hide something” and *ja:b* “bring”. In some cases, children display what might seem as complex constructs, which resemble much adult production, while, in practice, these forms emerge gradually, but not yet wholly, and do not yet match more complex abstract grammatical patterns, as used by adults, as if “they say the words, but they do not yet fully understand what they mean”. The semantic complexity limit is maintained in this age group even in the use of verbs which in adult language are semantically much more complex. One striking example, is that of the multi-argument verb *sa:*ʕ*ad* “help”, which involves the agent, the recipient, and most challenging—the action. Our corpus shows that children use this verb at 3–3.6, which is otherwise too early for such complexity. To account for this problematic piece of data, we resorted to the usage-based approach to language. The actual usage of *sa:*ʕ*ad* “help” does involve the child as agent and a parent as recipient, but no activity as the third argument of “help” is involved in any of this verb's occurrences in this age group, which is rather formulaic. In order to grasp what children actually mean when they supposedly “help mama” in the kitchen or “help papa” with the car, the examiner asked them specifically *ki:f b*
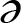
*tsa:*ʕ*edha* “how do you help her?”, and the answer was *bal*ʕ*ab ḥadha* “I play next to hers”; and to the examiner's question *ki:f b*
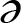
*tsa:*ʔ *ba:ba* “how do you help dad?” the answer was 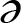
*lmatu:r ṣo:to* ʕ*a:li* “the motor was loud”. What these children mean when they “help” is that they spend time with a responsible adult, and the level of agency in their “help” is much lower than that of adults who really help someone in doing something. Low agency is indeed a primary behavioral property of young age, and this translates into a semantic property when using words which adults use with high agency. Similarly, while the verb *i*∫*ta*ḡ*al* ‘work' emerges at age 3–3.6, its meaning is much more limited in child language than in adult language, since in adult language it means that someone is actually doing some activity, while *i*∫*ta*ḡ*al* “work” in child language takes only a parent as its subject for “being away” as a state, not as an activity, with no further arguments as object or location; all that “work” really means in the younger ages is “be away”, or “be busy”, namely “unavailable *for me*”, as in “dad is working now”. According to our corpus, no typical complements for “work” in adult language have been found in the language of young children. In this respect, child-language “work” is neither transitive nor agentive, just stative, comparable to *ra:ḥ* “be away”. Similarly, the verb *t*ʕ*allam* “learn”, which emerges in age group 3–3.6, would at first sight seem semantically too complex for that age group with respect to the number of arguments: at this age the use of “learn” is not for a process of learning something new from someone else, perceiving a change state from having less knowledgeable having to more knowledgeable. This verb, as it is used in the corpus, is semantically much simpler: a child's input consists of adults' output about “learning at the kindergarten” and the child uses this verb initially in this collocation only, as if saying “learning at the kindergarten” meaning “going to the kindergarten”, and in fact just “being” there. So, what might seem as a highly transitive verb conveying some multi-argument complex activity in adult language is merely some variation of a low-agency verb “being at a place” in child language. By the same token, the verb *i*ʕ*tara* “buy” also emerges at age group 3–3.6, and would also seem at first sight too complex in view of the arguments it involves: the *buyer* goes to a *shop*, pays *money*, takes the *merchandise* and comes back *home*. In child language, however, its semantic structure is limited to *parents* or other significant adults as subject and *present, candy* or other child-relevant concrete arguments as object; no money or price or shop or going-and-coming-back components are involved. In this respect, “buy” in child language covers merely verbs like “get” in the sense of “fetch”, comparable to *ja:b* “bring”; one particular usage in our corpus illustrates the meaning of “buy” in child-usage: *mama bti*∫*trilna* ʔ*uxt* “mom is buying us a sister” referring to a pregnant mother who is supposed to “bring” a sister to the family, yet conceptualized in the child's usage by the semantic equivalent “buy”, i.e., much less complex than “buy” in adult language. What we infer from child-usage of “help,” “learn,” and “buy” is the precise gap between emergence at 3–3.6 age group and the process leading to final acquisition in the future, to match adult usage. The semantic gap between child usage and adult usage of seemingly identical items shows that emergence does not yet mean full acquisition, as child usage has not yet attained that of adults. (see Figure 5).

Based on our analysis presented in the previous paragraph, we suggest the above Figure 5 as a visual representation to illustrate the gradual process in which age predicts the level of agency and the number of morphemes. The intersection between the circles presents the overlap in the development of morphological complexity and semantic complexity, the middle arrow represents the development axis in relation to age, and the arrows below represent the morpho-semantic usage of the child and an adult, which can be differ in semantic usage Thus, as age rises, so do both other variables, namely agency and the number of morphemes. And the level of agency can be determined based on the *actual child-usage* of the verbs rather than typical adult-usage, which is characterized by higher agency.

As opposed to the simplification of verb meaning detailed above, truly complex verbs, not only in adult language, but also in child language, emerge at a later age. These include verbs like *ra:qab* “watch closely, inspect”, which emerges, from age 4-on; and from age 5-on verbs like *ista*ʕ*mal* “use”. Based on this evidence, we can observe a clear semantic development in the verb inventory of preschool children in correlation with age, regardless of morphology.

### 4.2. The relationship of verb patterns and roots in the emergence of Arabic verbs

As we have seen so far, surprisingly young children use verbs in morphologically complex patterns. This usage, however, does not necessarily mean they have productive *command* of the system. Just like the seemingly complex verb *semantics*, which appear to be much simpler in the younger groups, so is seemingly complex verb *morphology* much simpler than the morphological complexity of the non-basic patterns. In effect, at age 6 there is an increase in the use of non-singletons, i.e., more than one verb of the same root in different patterns. As shown in Table 6, by age 5.0 there is a dramatic increase in the use of non-singletons, almost doubling the number of singletons compared to 4-year-old, supporting the emergence of knowledge of the root as an independent linguistic unit. The data in the youngest age-group 2.6–3.0 displays only 3 non-singletons. These findings suggest that same-root verb productivity becomes fully operational at the age of 5.0 as children begin to use verb patterns as a morphological device, and this is likely to point to the transition from emergence of separate lexemes to emergence of the non-concatenative morphological system.

## 5. Conclusion

The development by age of the verb lexicon in SpA child language is constrained by parameters of semantic complexity. With the rising of semantic complexity by age comes also diversity in verb patterns. Yet, while the semantic complexity rises with age, the morphological complexity of verbs can only be positively identified when verb patterns become distinctive devices between same-root verbs, and this is fully developed later than the emergence of isolated verbs in diverse verb patterns. Our analysis suggests that while semantic constraints determine the emergence of singletons, morphological constraints determine the emergence of non-singletons. Their emergence in older age groups appears to indicate that the emergence of verb patterns as a morphological device occurs later than the emergence of singletons in “verb patterns” not yet ready for derivational productivity. The perception of verb patterns as abstract linguistic entities beyond the actual verbs is therefore attained later than the semantically-constrained verbs in earlier childhood. We therefore conclude that whereas semantic complexity obstructs verbs from emerging in the lexicon in younger age groups, morphological complexity constitutes no such obstruction, since their perception as morphological devices is attained later in acquisition.

## Data availability statement

The raw data supporting the conclusions of this article will be made available by the authors, upon request, without undue reservation.

## Ethics statement

The studies involving human participants were reviewed and approved by Bar-Ilan University, Faculty of Humanities Institutional Review Board. Written informed consent to participate in this study was provided by the participants' legal guardian/next of kin.

## Author contributions

NT-M, SA-L, and ES-H contributed to conception and design of the study. NT-M organized the database and wrote the first draft of the manuscript. SA-L and ES-H performed the statistical analysis. All authors contributed to manuscript revision, read, and approved the submitted version.

## References

[B1] Ashkenazi O. (2015). Input–output relations in the early acquisition of Hebrew verbs (doctoral dissertation). Tel Aviv University, Tel Aviv, Israel.

[B2] Ashkenazi O. Ravid D. Gillis S. (2016). Breaking into the Hebrew verb system: a learning problem. First Lang. 36, 505–524. 10.1177/0142723716648865

[B3] Berman R. A. (1993). “Crosslinguistic perspectives on native language acquisition,” in Progression and Regression in Language: Sociocultural, Neuro-psychological, and Linguistic Perspectives, eds. K. Hyltenstam and A. Viberg (Cambridge: Cambridge University Press), 245–266.

[B4] Berman R. A. (2004). “Between emergence and mastery: the long developmental route of language acquisition,” in Language Development Across Childhood and Adolescence, eds. R. A. Berman, (Amsterdam: John Benjamins), 9–34.

[B5] Berman R. A. Dromi E. (1982). A metamorphic measure of early language development: data from modern Hebrew. J. Child Lang. 9, 403–424. 10.1017/S0305000900004785 7119042

[B6] Berman R. A. Dromi E. (1984). On marking time without aspect in child language. Papers Rep. Child Lang. Dev. 23, 21–32.

[B7] Boudelaa S. (2014). “Is the Arabic mental lexicon morpheme-based or stem-based? Implications for spoken and written word recognition,” *in Handbook of Arabic Literacy*, (Dordrecht: Springer), 31–54.

[B8] Budwig N. (1995). A Developmental-Functionalist Approach to Child Language. Mahwah, NJ: Lawrence Erlbaum Associates.

[B9] Bybee J. (1995). Regular morphology and the lexicon. Lang. Cogn. Proc. 10, 425–455. 10.1080/01690969508407111

[B10] Bybee J. (2006). From usage to grammar: the mind's response to repetition. Language 82, 711–733. 10.1353/lan.2006.0186

[B11] Cenko E. Budwig N. (2007). “The acquisition of early verb constructions in Albanian: a first look at transitives and intransitives,” in A Supplement to the Proceedings of the 31st Boston University Conference on Language. Available online at: https://www.researchgate.net/profile/Enila-Cenko/publication/255598246_The_acquisition_of_early_verb_constructions_in_Albanian_A_first_look_at_transitives_and_intransitives/links/566bdf5c08ae430ab4fc28fe/The-acquisition-of-early-verb-constructions-in-Albanian-A-first-look-at-transitives-and-intransitives.pdf (accessed April 18, 2022).

[B12] Dattner E. Ashkenazi O. Ravid D. Levie R. (2022). Explaining dynamic morphological patterns in acquisition using Network Analysis. Morphology. 1–46. 10.1007/s11525-022-09394-0

[B13] Fassi Fehri A. (1994). “Configurations and transitivity splits in the Arabic lexicon,” in Configurations, eds A. Di Sciulo (Somervilee: Cascadilla Press), 51–78.

[B14] Gennari S. Poeppel D. (2003). Processing correlates of lexical semantic complexity. Cognition 89, B27–41. 10.1016/S0010-0277(03)00069-6 12893127

[B15] Gentner D. (1978). On relational meaning: the acquisition of verb meaning. Child Dev. 49, 988–998. 10.2307/1128738

[B16] Gentner D. (1982). Why Nouns are Learned Before Verbs: Linguistic Relativity Versus Natural Partitioning. Center for the Study of Reading Technical Report; no. 257.

[B17] Glanville P. (2011). The Arabic verb root and stem and their contribution to verb meaning (doctoral dissertation). The University of Texas at Austin, Austin, TX, United States.

[B18] Golinkoff R. M. Hirsh-Pasek K. (2006). “Introduction: Progress on the verb learning front,” in Action Meets Word: How Children Learn Verbs (Oxford University Press), 3–28.

[B19] Golinkoff R. M. Hirsh-Pasek K. (2008). How toddlers begin to learn verbs. Trend. Cogn. Sci. 12, 397–403. 10.1016/j.tics.2008.07.003 18760656

[B20] Guerssel M. Lowenstamm J. (1996). “Ablaut in Classical Arabic measure I active verbal forms,” in Studies in Afroasiatic Grammar, eds. J. Lecarme, J. Lowenstam and U. Shlonsky, (The Hague: Holland Academic Graphics), 123–134.

[B21] Hallman P. (2006). Causativity and Transitivity in Arabic. Available online at: http://site.iugaza.edu.ps/wamer/files/2019/02/Causativity-and-Transitivity-in-Arabic.pdf (accessed April 18, 2023).

[B22] Jastrow O. (2004). The Arabic dialects of the Muthallath (Central Israel). Jerusalem Stud. Arabic Islam 29, 166–176.25324089

[B23] Kibrik A. (2012). “What's in the head of head-marking languages”, in *Argument Structure and Grammatical: A Cross-Linguistic Typology*. ed. P. Suihkonen, B. Comrie and V. Solovyev (Amsterdam; Philadelphia, PA: John Benjamins), 211–240.

[B24] Koptjevskaja-Tamm M. (2012). New directions in lexical typology. Linguistics 50, 373–394. 10.1515/ling-2012-0013

[B25] Laks L. Hamad I. Saiegh Haddad E. (2019). Verbal patterns in Palestinian Arabic. Mental Lexicon 14, 209–236. 10.1075/ml.00005.lak

[B26] Lenth R. (2019). emmeans: Estimated Marginal Means, aka Least-Squares Means. R package version 1.3.3.

[B27] Levie R. Ashkenazi O. Eitan Stanzas S. Zwilling R. Raz E. Hershkovitz L.. (2020). The route to the derivational verb family in Hebrew: a psycholinguistic study of acquisition and development. Morphology 30, 1–60. 10.1007/s11525-020-09348-4

[B28] Lüdecke D. (2021). sjPlot: Data Visualization for Statistics in Social Science. R package version 2.8.10.

[B29] McCarthy J. (1981). A prosodic theory of nonconcatenative morphology. Linguistic Inquiry 12, 373–418.15019552

[B30] Nelson K. (1973). Structure and strategy in learning to talk. Monogr. Soc. Res. Child Dev. 38, 1–135. 10.2307/1165788

[B31] Ouhalla J. (2014). “Causatives, anticausatives and lexicalization,” in The Form of Structure, the Structure of Form: Essays in honor of Jean Lowenstamm, ed. S. Benjaballah, N. Faust, M. Lahrouchi and N. Lampitelli (Amsterdam: John Benjamins), 333–348.

[B32] Ravid D. (1995). Language Change in Child and Adult Hebrew: A Psycholinguistic Perspective. New York, NY: Oxford University Press.

[B33] Ravid D. (2019). “First-language acquisition of morphology,” in Oxford Research Encyclopedia, Linguistics, ed. M. Aronoff (Oxford: Oxford University Press).

[B34] Ryding K. C. (2005). A Reference Grammar of Modern Standard Arabic. Cambridge: Cambridge University Press.

[B35] Shalhoub-Awwad Y. (2020). The role of nominal word pattern in Arabic reading acquisition: insights from cross-modal priming. Sci. Stud. Read. 24, 307–320. 10.1080/10888438.2019.1668396

[B36] Shalhoub-Awwad Y. Leikin M. (2016). The lexical status of the root in processing morphologically complex words in Arabic. Sci. Stud. Read. 20, 296–310. 10.1080/10888438.2016.1180525

[B37] Talmy L. (1985). “Lexicalization patterns: Semantic structure in lexical forms,” in Language Typology and Syntactic Description, ed. T. Shopen (Cambridge: Cambridge University Press), 36–149.

[B38] Team R. C. R Core Team. (2012). R: A Language and Environment for Statistical Computing. R Foundation for Statistical Computing, Vienna, Austria.

[B39] Tomasello M. (2003). Constructing a Language: A Usage-Based Theory of Language Acquisition. Cambridge, MA: Harvard University Press.

[B40] Wittig S. (1990). Valence patterns and sentence structures of Arabic functional verb complexes-syntactic analysis. J. Arabic Linguist. 2, 17–29.

[B41] Younes M. (2000). “Redundancy and productivity in Palestinian Arabic verb derivation,” in Proceedings of the Third International Conference of AÏDA, ed. M. Mifsud (Malta: Salesian Press), 27–32.

