# Supplementary material for: Emergence of verb-pattern morphology in young Arabic speakers: morphological and semantic features
Source: Front Psychol. 2023 May 12;14:1127640. doi: 10.3389/fpsyg.2023.1127640 (PMC10213558; doi:10.3389/fpsyg.2023.1127640)
Supplement: Supplementary file 1 [file Data_Sheet_1.docx]

Supplementary Material

Emergence of verb-pattern morphology in young Arabic speakers: Morphological and semantic features

**Appendix A.** Description of Study Sample (age groups)

|  | Age | Mean (SD) | N (Female) |
| --- | --- | --- | --- |
| I | 2.6-3 | 2.8 (0.11) | 17 (7) |
| II | 3-3.6 | 3.3(0.24 | 20(10) |
| III | 3.6-4 | 3.8 (0.13) | 17(12) |
| IV | 4-4.6 | 4.3 (0/13) | 20 (10) |
| V | 4.6-5 | 4.8(0.17) | 19(12) |
| VI | 5-5.6 | 5.4 (0.17) | 19(8) |
| VII | 5.6-6 | 5.8 (0.08 | 21(10) |
| Total |  |  | 133 (69) |

**Appendix B**. Estimates of the model predicting the proportion of verbal patterns – tokens

|  | **perc** | | |
| --- | --- | --- | --- |
| *Predictors* | *Estimates* | *CI* | *p* |
| (Intercept) | 0.67 | 0.65 – 0.69 | **<0.001** |
| Age3;0-3;6 | -0.03 | -0.06 – -0.00 | **0.026** |
| Age3;6-4;0 | 0.00 | -0.03 – 0.03 | 0.976 |
| Age4;0-4;6 | 0.01 | -0.02 – 0.03 | 0.644 |
| Age4;6-5;0 | -0.03 | -0.06 – -0.01 | **0.015** |
| Age5;0-5;6 | -0.05 | -0.08 – -0.02 | **<0.001** |
| Age5;6-6;0 | -0.01 | -0.04 – 0.02 | 0.444 |
| Pattern [fa''al] | -0.39 | -0.42 – -0.37 | **<0.001** |
| Pattern [fa:'al] | -0.64 | -0.67 – -0.61 | **<0.001** |
| Pattern [fa'lal] | -0.64 | -0.67 – -0.62 | **<0.001** |
| Pattern [tfa''al] | -0.58 | -0.60 – -0.55 | **<0.001** |
| Pattern [infa'al] | -0.65 | -0.68 – -0.62 | **<0.001** |
| Pattern [af'al] | -0.65 | -0.68 – -0.63 | **<0.001** |
| Pattern [ifta'al] | -0.66 | -0.69 – -0.64 | **<0.001** |
| Age3;0-3;6: Pattern fa''al | -0.02 | -0.06 – 0.02 | 0.255 |
| Age3;6-4;0: Pattern fa''al | -0.12 | -0.16 – -0.08 | **<0.001** |
| Age4;0-4;6: Pattern fa''al | -0.13 | -0.16 – -0.09 | **<0.001** |
| Age4;6-5;0: Pattern fa''al | -0.06 | -0.10 – -0.02 | **0.002** |
| Age5;0-5;6: Pattern fa''al | 0.02 | -0.02 – 0.05 | 0.399 |
| Age5;6-6;0: Pattern fa''al | -0.07 | -0.10 – -0.03 | **<0.001** |
| Age3;0-3;6: Pattern fa:'al | 0.03 | -0.00 – 0.07 | 0.087 |
| Age3;6-4;0: Pattern fa:'al | 0.00 | -0.04 – 0.04 | 0.987 |
| Age4;0-4;6:Patternfa:'al | -0.02 | -0.05 – 0.02 | 0.384 |
| Age4;6-5;0:Patternfa:'al | 0.04 | -0.00 – 0.07 | 0.061 |
| Age5;0-5;6:Patternfa:'al | 0.03 | -0.00 – 0.07 | 0.079 |
| Age5;6-6;0:Patternfa:'al | -0.01 | -0.05 – 0.03 | 0.533 |
| Age3;0-3;6:Patternfa'lal | 0.05 | 0.01 – 0.08 | **0.011** |
| Age3;6-4;0:Patternfa'lal | -0.00 | -0.04 – 0.03 | 0.860 |
| Age4;0-4;6:Patternfa'lal | -0.01 | -0.05 – 0.02 | 0.488 |
| Age4;6-5;0:Patternfa'lal | 0.03 | -0.01 – 0.06 | 0.159 |
| Age5;0-5;6:Patternfa'lal | 0.06 | 0.02 – 0.09 | **0.002** |
| Age3;0-3;6:Patterntfa''al | -0.05 | -0.08 – -0.01 | **0.011** |
| Age3;6-4;0:Patterntfa''al | -0.04 | -0.08 – -0.00 | **0.030** |
| Age4;0-4;6:Patterntfa''al | -0.04 | -0.08 – -0.01 | **0.015** |
| Age4;6-5;0:Patterntfa''al | 0.01 | -0.03 – 0.04 | 0.774 |
| Age5;0-5;6:Patterntfa''al | 0.00 | -0.03 – 0.04 | 0.814 |
| Age3;0-3;6:Patterninfa'al | 0.02 | -0.02 – 0.06 | 0.310 |
| Age3;6-4;0:Patterninfa'al | 0.00 | -0.04 – 0.04 | 0.949 |
| Age4;0-4;6:Patterninfa'al | 0.00 | -0.04 – 0.04 | 0.951 |
| Age4;6-5;0:Patterninfa'al | 0.04 | 0.00 – 0.08 | **0.049** |
| Age5;0-5;6:Patterninfa'al | 0.05 | 0.01 – 0.09 | **0.009** |
| Age5;6-6;0:Patterninfa'al | 0.01 | -0.03 – 0.05 | 0.613 |
| Age3;0-3;6:Patternaf'al | 0.04 | 0.00 – 0.07 | **0.041** |
| Age3;6-4;0:Patternaf'al | 0.01 | -0.03 – 0.04 | 0.768 |
| Age4;0-4;6:Patternaf'al | -0.00 | -0.04 – 0.03 | 0.807 |
| Age4;6-5;0:Patternaf'al | 0.03 | -0.01 – 0.07 | 0.101 |
| Age5;0-5;6:Patternaf'al | 0.04 | 0.01 – 0.08 | **0.021** |
| Age3;0-3;6:Patternifta'al | 0.03 | -0.00 – 0.07 | 0.079 |
| Age3;6-4;0:Patternifta'al | 0.01 | -0.02 – 0.05 | 0.452 |
| Age4;0-4;6:Patternifta'al | 0.01 | -0.02 – 0.05 | 0.489 |
| Age4;6-5;0:Patternifta'al | 0.05 | 0.01 – 0.09 | **0.013** |
| Age5;0-5;6:Patternifta'al | 0.06 | 0.02 – 0.10 | **0.001** |
| Age5;6-6;0:Patternifta'al | 0.01 | -0.02 – 0.05 | 0.491 |

Note: the reference category for Age group is 2;6-3;0; the reference category for Pattern is fa’al

**Appendix C**. *GLM model summary for age x pattern*

| *Predictors* | *Incidence Rate Ratios* | *CI* | *p* |
| --- | --- | --- | --- |
| (Intercept) | 31.56 | 23.95 – 40.70 | **<0.001** |
| 3;0-3;6 | 2.56 | 1.90 – 3.50 | **<0.001** |
| 3;6-4;0 | 2.39 | 1.76 – 3.27 | **<0.001** |
| 4;0-4;6 | 2.88 | 2.14 – 3.92 | **<0.001** |
| 4;6-5;0 | 3.46 | 2.59 – 4.68 | **<0.001** |
| 5;0-5;6 | 4.14 | 3.13 – 5.58 | **<0.001** |
| 5;6-6;0 | 4.30 | 3.25 – 5.79 | **<0.001** |
| fa''al | 0.49 | 0.43 – 0.56 | **<0.001** |
| fa:'al | 0.06 | 0.04 – 0.08 | **<0.001** |
| fa'lal | 0.05 | 0.03 – 0.06 | **<0.001** |
| tfa''al | 0.08 | 0.06 – 0.10 | **<0.001** |
| infa'al | 0.07 | 0.05 – 0.09 | **<0.001** |
| af'al | 0.04 | 0.02 – 0.05 | **<0.001** |
| ifta'al | 0.03 | 0.02 – 0.04 | **<0.001** |

**Appendix D**. Model estimates predicting probability of using verb types

| *Predictors* | *Odds Ratios* | *CI* | *p* |
| --- | --- | --- | --- |
| (Intercept) | 0.18 | 0.12 – 0.26 | **<0.001** |
| Age Group 3;0-3;6 | 5.02 | 3.36 – 7.52 | **<0.001** |
| Age Group 3;6-4 | 4.00 | 2.68 – 5.99 | **<0.001** |
| Age Group 4-4;6 | 6.02 | 4.02 – 9.03 | **<0.001** |
| Age Group 4;6-5 | 10.24 | 6.75 – 15.56 | **<0.001** |
| Age Group 5-5;6 | 22.59 | 14.22 – 35.88 | **<0.001** |
| Age Group 5;6-6 | 30.27 | 18.55 – 49.40 | **<0.001** |
| Agentivity | 0.16 | 0.07 – 0.33 | **<0.001** |
| Age Group 3;0-3;6 * Agentivity | 0.27 | 0.10 – 0.71 | **0.008** |
| Age Group 3;6-4* Agentivity | 0.43 | 0.17 – 1.09 | 0.076 |
| Age Group 4-4;6* Agentivity | 0.37 | 0.15 – 0.91 | **0.031** |
| Age Group 4;6-5* Agentivity | 0.28 | 0.11 – 0.68 | **0.005** |
| Age Group 5-5;6 * Agentivity | 0.16 | 0.06 – 0.38 | **<0.001** |
| Age Group 5;6-6* Agentivity | 0.12 | 0.05 – 0.29 | **<0.001** |
| **Random Effects** | | | |
| σ^2^ | 3.29 | | |
| τ_00_ _Pattern_ | 0.08 | | |
| ICC | 0.02 | | |
| N _Pattern_ | 12 | | |
| Observations | 3514 | | |
| Marginal R^2^ / Conditional R^2^ | 0.472 / 0.485 | | |

**Appendix E**. Estimates of GLM model predicting probability of producing verb types

| *Predictors* | *Odds Ratios* | *CI* | *p* |
| --- | --- | --- | --- |
| (Intercept) | 0.15 | 0.10 – 0.22 | **<0.001** |
| Age Group 3;0-3;6 | 2.08 | 1.31 – 3.29 | **0.002** |
| Age Group 3;6-4;0 | 1.78 | 1.12 – 2.84 | **0.015** |
| Age Group 4;0-4;6 | 2.36 | 1.50 – 3.71 | **<0.001** |
| Age Group 4;6-5;0 | 2.76 | 1.76 – 4.32 | **<0.001** |
| Age Group 5;0-5;6 | 3.88 | 2.50 – 6.02 | **<0.001** |
| Age Group 5;6-6;0 | 4.01 | 2.58 – 6.23 | **<0.001** |
| Transitivity | 0.59 | 0.34 – 1.04 | 0.069 |
| Age Group 3;0-3;6 * Transitivity | 2.46 | 1.24 – 4.89 | **0.010** |
| Age Group 3;6-4;0* Transitivity | 2.77 | 1.38 – 5.54 | **0.004** |
| Age Group 4;0-4;6* Transitivity | 2.73 | 1.38 – 5.40 | **0.004** |
| Age Group 4;6-5;0* Transitivity | 3.49 | 1.78 – 6.85 | **<0.001** |
| Age Group 5;0-5;6* Transitivity | 3.39 | 1.73 – 6.62 | **<0.001** |
| Age Group 5;6-6;0 * Transitivity | 3.68 | 1.88 – 7.20 | **<0.001** |
| **Random Effects** | | | |
| σ^2^ | 3.29 | | |
| τ_00_ _Type_ | 0.03 | | |
| ICC | 0.01 | | |
| N _Type_ | 8 | | |
| Observations | 3444 | | |
| Marginal R^2^ / Conditional R^2^ | 0.130 / 0.137 | | |

**Appendix F**. Estimates of GLM model predicting probability of producing verb types

| *Predictors* | *Odds Ratios* | *CI* | *p* |
| --- | --- | --- | --- |
| (Intercept) | 0.06 | 0.04 – 0.08 | **<0.001** |
| Age Group 3;0-3;6 | 3.22 | 2.30 – 4.50 | **<0.001** |
| Age Group 3;6-4;0 | 2.89 | 2.06 – 4.05 | **<0.001** |
| Age Group 4;0-4;6 | 3.80 | 2.72 – 5.30 | **<0.001** |
| Age Group 4;6-5;0 | 5.10 | 3.67 – 7.08 | **<0.001** |
| Age Group 5;0-5;6 | 6.92 | 5.00 – 9.58 | **<0.001** |
| Age Group 5;6-6;0 | 7.39 | 5.34 – 10.22 | **<0.001** |
| Reciprocal | 0.02 | 0.01 – 0.04 | **<0.001** |
| Reflexive | 0.18 | 0.14 – 0.23 | **<0.001** |
| Inchoative | 2.67 | 2.29 – 3.11 | **<0.001** |
| **Random Effects** | | | |
| σ^2^ | 3.29 | | |
| τ_00_ _Pattern_ | 0.02 | | |
| ICC | 0.01 | | |
| N _Pattern_ | 12 | | |
| Observations | 7028 | | |
| Marginal R^2^ / Conditional R^2^ | 0.530 / 0.533 | | |

**Appendix G**.

| Analysis | R script |
| --- | --- |
| LMM (tokens) | model1.0 <- lmer(perc ~ 1+ (1 \| Name), data=Naila_data2long2, REML=FALSE)  model1.1 <- update(model1.0, .~.+ Age)  model1.2 <- update(model1.1, .~.+ Pattern)  model1.3 <- update(model1.2, .~.+ Age*Pattern)  anova(model1.0, model1.1,model1.2,model1.3) |
| GLM (types) | model_type0 <- glm(score.sum ~ 1, data=types_long_s,family="poisson"(link = "log"))  model_type1 <- update(model_type0, .~.+ age_gr_col)  model_type2 <- update(model_type1, .~.+ Pattern)  model_type3 <- update(model_type2, .~.+ age_gr_col * Pattern)  anova(model_type0,model_type1,model_type2,model_type3) |
| GLMM  (sematic types) | Stative vs. agentive:  modelSAG <- glmer(freq ~ 1 + (1 \| Binyan), data=types_verbs_act_state.merge3,family="binomial")  modelSAG.1 <- update(modelSAG, .~.+ age_gr)  modelSAG.2 <- update(modelSAG.1, .~.+ act_state)  modelSAG.3 <- update(modelSAG.2, .~.+ age_gr*act_state)  anova(modelSAG,modelSAG.1,modelSAG.2,modelSAG.3)  Transitive vs intransitive:  modelTR.0 <- glmer(freq ~ 1 + (1 \| item), data=types_verbs_trans_intr.merge3,family="binomial")  modelTR.1 <- update(modelTR.0, .~.+ age_gr)  modelTR.2 <- update(modelTR.1, .~.+ trans_intr1)  modelTR.3 <- update(modelTR.2, .~.+ age_gr*trans_intr1)  anova(modelTR.0,modelTR.1,modelTR.2,modelTR.3)  Semantic categories:  modelSEM.0 <- glmer(freq ~ 1 + (1 \| Pattern), data=types_verbs_04.merge3,family="binomial")  modelSEM.1 <- update(modelSEM.0, .~.+ age_gr)  modelSEM.2 <- update(modelSEM.1, .~.+ type_4.1)  modelSEM.3 <- update(modelSEM.2, .~.+ age_gr*type_4.1)  anova(modelSEM.0,modelSEM.1,modelSEM.2,modelSEM.3) |
